# An Innovative Bioremediation Approach to Heavy Metal Removal: Combined Application of *Chlorella vulgaris* and Amine-Functionalized MgFe_2_O_4_ Nanoparticles in Industrial Wastewater Treatment

**DOI:** 10.3390/ijms26125467

**Published:** 2025-06-07

**Authors:** Tímea Fóris, Péter Koska, Ágnes Maria Ilosvai, Ferenc Kristály, Lajos Daróczi, László Vanyorek, Béla Viskolcz

**Affiliations:** 1 Institute of Chemistry, University of Miskolc, 3515 Miskolc-Egyetemváros, Hungary; timea.foris1@uni-miskolc.hu (T.F.); peter.zoltan.koska@uni-miskolc.hu (P.K.); askkf@uni-miskolc.hu (Á.M.I.); laszlo.vanyorek@uni-miskolc.hu (L.V.); 2 Higher Education and Industrial Cooperation Centre, University of Miskolc, 3515 Miskolc-Egyetemváros, Hungary; 3 Institute of Mineralogy and Geology, University of Miskolc, 3515 Miskolc-Egyetemváros, Hungary; ferenc.kristaly@uni-miskolc.hu; 4 Department of Solid State Physics, University of Debrecen, P.O. Box 2, 4010 Debrecen, Hungary; lajos.daroczi@science.unideb.hu

**Keywords:** *Chlorella vulgaris*, heavy metal adsorption, amine-functionalized magnesium ferrite

## Abstract

The removal of heavy metals from industrial wastewater remains a major environmental challenge, demanding efficient, sustainable solutions. This study explores the combined use of *Chlorella vulgaris* and amine-functionalized magnesium ferrite (MgFe_2_O_4_-NH_2_) nanoparticles to remove cobalt ions from battery effluents. The research aims to explore the capacity of *C. vulgaris* to adsorb heavy metals, followed by their separation using magnetic nanoparticles. Cobalt adsorption by *C. vulgaris* was facilitated through the interaction of metal ions on the cell wall, achieving a removal efficiency of 96.44% within 30 min, which increased to 98.78% over 10 h. Amine-functionalized MgFe_2_O_4_ nanoparticles, synthesized and characterized using HRTEM, FTIR, and VSM, displayed high surface reactivity due to the presence of -NH_2_ and -OH groups. At neutral pH, zeta potential measurements revealed a slightly negative charge (−5.6 ± 4.3 mV), while protonation at lower pH levels enhanced electrostatic interactions with the negatively charged algal biomass. Magnetic separation of the cobalt-adsorbed biomass achieved efficiencies ranging from 94.9% to 99.2% within 60 s, significantly outperforming conventional sedimentation methods. SEM and FTIR analyses confirmed the binding of nanoparticles to algal cell walls. The even distribution of MgFe_2_O_4_ nanoparticles on algal surfaces was further validated by TEM imaging, and the strong magnetic properties of the nanoparticles enabled rapid and efficient separation under an external magnetic field.

## 1. Introduction

The contamination of natural waters and soil with heavy metals from industrial effluents represents a significant environmental concern in the present era. The battery industry represents a significant source of heavy metal contamination, with industrial wastewater from battery manufacturing and recycling companies containing considerable quantities of heavy metals [1]. The cathodes of NMC (nickel manganese cobalt oxide) lithium-ion batteries most used in the automotive industry typically contain nickel, cobalt, and manganese in a ratio of 1:1:1 or 5:3:2. The cathode of the also commonly used NCA (lithium nickel cobalt aluminum oxide) batteries has a manganese content of 82% and a cobalt content of 12% [2,3]. At higher concentrations, heavy metal pollution is dangerous because of its direct toxic effects on living organisms; however, at low concentrations it also poses a serious risk due to its accumulation in the food chain [4]. Several technologies have been developed to remove heavy metals from industrial wastewater, including ion exchange, reverse osmosis, electrodialysis and ultrafiltration [5]. However, in addition to their efficiency, these processes often present several disadvantages, including incomplete metal removal, sludge formation, high reagent and energy requirements, metal precipitate aggregation, and fouling of membrane filters. In consequence, alternative technologies have emerged as a significant area of research in recent years. Among these, bioremediation processes have attracted particular attention as a treatment technology for the removal of heavy metals [6]. This interest stems from technology’s perceived economic feasibility, efficiency, and environmental compatibility [7].

Bioremediation processes encompass technologies that utilize biological systems to diminish the concentration and detrimental effects of pollutants to an acceptable level. The main organisms used in bioremediation technologies are microorganisms, including bacteria, algae, and yeasts [8]. Algal cells are capable of adsorbing toxic metal ions onto their cell surface, followed by intracellular accumulation through binding to phytochelatins and metallothioneins, and subsequent sequestration in large quantities within the vacuole [9]. *Chlorella vulgaris*, a freshwater microalga, has been identified as a particularly efficacious agent in this regard [10]. The cell wall of *Chlorella vulgaris* is composed of complex carbohydrates, with a network of cellulose fibres attached to a variety of polysaccharides, including hemicellulose and glycoproteins [11]. Furthermore, monosaccharides (e.g., glucose, mannose, galactose, xylose, fucose, arabinose), lipids, and considerable quantities of glucosamines are present. The functional groups present in these polymer molecules are significant regarding the processes of adsorption. The cell wall biopolymer was found to contain several functional groups, including carboxyl (-COOH), hydroxyl (-OH), amino (-NH_2_), carbonyl (-C=O), ester (-CO-O), sulfhydryl (-SH), sulphate (-SO_4_^2−^), and phosphate (PO_4_^3−^). The functional groups are protonated in accordance with the pH level, thereby influencing the charge of the cell wall [12]. It can be observed that above pH 3, the negative charge of carboxyl groups, phosphate groups, and hydroxyl groups is predominant, with positively charged heavy metal cations adsorbing to the negatively charged cell wall [13]. The adsorption of cell surface biopolymers can occur by several mechanisms, including physisorption, ion exchange, chelation, and complexation [14]. The biosorption capacity of the microalga *Chlorella vulgaris* has been the subject of extensive research in relation to various heavy metal ions, including Cu^2+^, Pb^2+^, Ni^2+^, Zn^2+^ and Hg^2+^ [15,16]. However, little data is available on the adsorption efficiency of Co^2+^ ions, mainly from wastewater generated during battery recycling processes [17].

In addition to the removal of heavy metal ions from industrial wastewater, the issue of how to remove algae that accumulate heavy metal ions from wastewater must be addressed. Several conventional methods have been developed for the removal of algae from wastewater. The most used methods are sedimentation, flotation, coagulation and flocculation, filtration, and sedimentation [18]. Magnetic separation is an innovative technology that is characterized by rapidity, cost-effectiveness, and energy efficiency. Algal cells exhibit a high affinity for the adsorption of magnetisable nanoparticles (MNPs) onto the surface of their cell walls, thereby enabling their isolation from wastewater in a magnetic field of sufficient strength [19,20]. The process of magnetic separation of green algae was conducted within a 1 L graduated cylinder under optimal conditions. The utilization of magnetite powder and a neodymium magnet ensured the effective removal of algae within 30 s, whereas conventional sedimentation methods necessitated over two hours for the same process [21]. Both naked and surface-functionalized (using polyacrylamides, chitosan, poly diallyldimethylammonium chloride (PDDA), aminoclay, polyethylenimine (PEI), 3-aminopropyl triethoxysilane (APTES)) magnetic nanoparticles have been applied for the harvesting of *C. vulgaris* [22,23,24,25,26,27]. Specifically, amine-functionalized MgFe_2_O_4_ nanoparticles exhibit high efficiency in binding to cell walls rich in negatively charged carboxyl groups [28]. Furthermore, magnetic biomass can be rapidly and efficiently separated by applying an external magnetic field, facilitating streamlined recovery and reuse.

The purpose of this study is to examine the capacity of *Chlorella vulgaris* cells to adsorb cobalt ions, which are heavy metal ions that may be present in effluents from battery manufacturing processes. This study further investigates the capacity of cobalt-adsorbed algal cells to take up amine-functionalized MgFe_2_O_4_ nanoparticles onto their cell walls and to facilitate sedimentation within a magnetic field. This approach could enable the development of an innovative and environmentally friendly method for the removal of heavy metals from industrial wastewater in a more cost-effective manner, thereby complementing conventional wastewater treatment technologies. The interactions between microalgal cells and magnetic nanoparticles were analyzed by scanning electron microscopy (SEM), with particular attention to identifying the functional groups involved by Fourier transform infrared spectroscopy (FTIR) measurements. The particle size and morphology of the magnetic nanoparticles were characterized by HRTEM. Additionally, zeta potential measurements were conducted to examine the separation mechanism of *C. vulgaris* microalgae facilitated by synthesized iron oxide magnetic particles.

## 2. Results

### 2.1. Characterization of the Amine-Functionalized Magnesium Ferrite Nanoparticles

In addition to the surface polarity, the sorption capacity of the adsorbents is highly dependent on their specific surface area and, therefore, a specific surface area (SSA) determination was performed. Consequently, CO_2_ adsorption–desorption measurements were conducted at 273 K in accordance with the Dubinin–Astakhov (DA) method to ascertain SSA. The surface area of the MgFe_2_O_4_-NH_2_ sample was found to be 54.4 m^2^ g^−1^, a value that is comparable to previously reported results (SABET: 47 m^2^ g^−1^ and 56.5 m^2^ g^−1^) [29].

**Figure 1:**
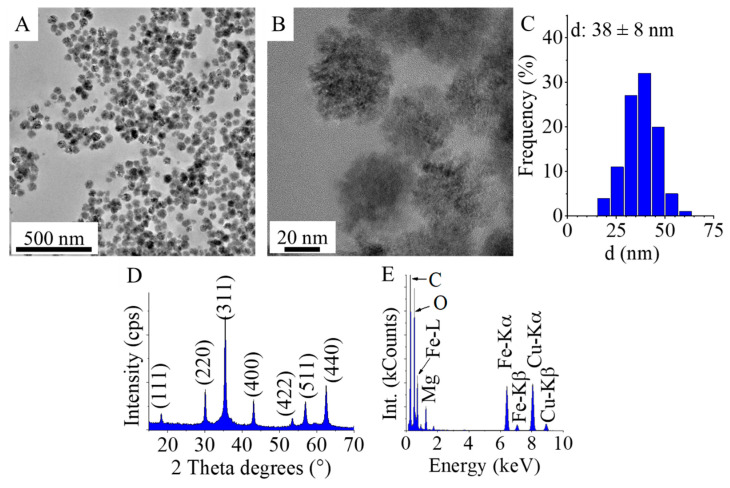
HRTEM pictures (**A**,**B**), size distribution (**C**), SAED picture (**D**), and EDS (**E**) of the amine-functionalized MgFe_2_O_4_ nanoparticles.

Particle size and the morphology of the magnetic nanoparticles were examined by HRTEM. On the HRTEM images, spherical ferrite nanoparticles are visible; these aggregated spheres build up from individual, 7–10 nm size nanoparticles (Figure 1A,B). The particle size histogram was made based on the HRTEM images (by using scalebar) of the amine-functionalized MgFe_2_O_4_ samples using ImageJ image analyser software (Available at: https://imagej.net/ij/download.html, accessed on 15 April 2022.) (Figure 1C). The average diameter of the aggregated spheres is 38 ± 8 nm. The selected area electron diffraction (SAED) technique was used to identify the individual particles (Figure 1D). The d-spacing values obtained from the evaluation of the SAED images show excellent correlation with the lattice plane spacings determined for magnesium ferrite based on an X-ray database (PDF 36-0398). To confirm that no other crystalline phases next to magnesium ferrite were present in the sample, XRD measurements were performed. Only reflections characteristic of MnFe_2_O_4_ spinel were observed on the XRD diffractogram. The Miller indices associated with the reflections were naturally the same as those marked on the SAED image. The XRD pattern of the sample (Figure 1D) revealed peaks of only magnesium ferrite, located at (2 Th/hkl) 18.2°/(111), 30.1°/(220), 35.4°/(311), 43.1°/(400), 53.4°/(422), 56.9°/(511) and 62.6°/(440), matching to PDF 36–0398 card. Other phases as residue were not detected; therefore, it can be stated that the synthesis method was applicable preparation of pure spinel phase nanoparticles. In the energy dispersive X-ray (EDS) spectrum, bands can be identified that correspond to the chemical elements that make up magnesium ferrite, namely oxygen, iron and magnesium (Figure 1E). The presence of copper and carbon in the EDS image can be explained by the presence of these elements in the sample holder material (copper grid, lacey carbon). The presence of carbon is also due to the adsorption of the organic compounds used in the synthesis (ethylene glycol and monoethanol amine) on the surface of the particles.

**Figure 2:**
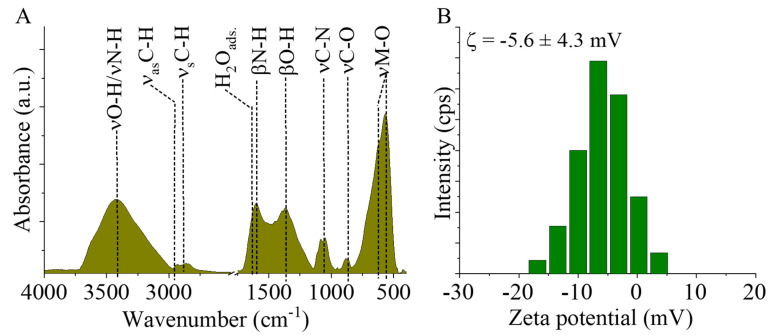
FTIR spectra (**A**) and zeta potential distribution diagram (**B**) of the MgFe_2_O_4_-NH_2._

To understand the adsorption interactions between algae and magnetic nanoparticles, it is important to identify the functional groups on the surface of the particles; thus, Fourier transformation infrared spectroscopic measurement was carried out to confirm the presence of the -NH_2_ and -OH groups. Two characteristic bands are observed on the spectrum of MgFe_2_O_4_ at 564 cm^−1^ and 621 cm^−1^ wavenumbers, which are assigned to intrinsic stretching vibration modes of the metal–oxygen bonds (νM-O) at the octahedral and tetrahedral sites [30] (Figure 2A). The bands corresponding to the stretching vibration modes of C-N and C-O bonds at 1052 cm^−1^ and 876 cm^−1^ wavenumbers, which are originated from the amine (-NH_2_) and hydroxyl (-OH) groups. Another absorption band was identified at 1365 cm^−1^ which can belong to the OH bending vibrations. The band of bending vibration mode of the N–H bonds was found at 1610 cm^−1^ wavenumber, corresponding to the free amine functional groups. moreover the presence of adsorbed water molecules may also contribute significantly to this band as shoulder at 1638 cm^−1^. The symmetric and asymmetric stretching vibration of the aliphatic and aromatic C-H bonds resulted in absorption at 2870 cm^−1^ and 2941 cm^−1^, which can be explained by the adsorbed organic molecules (EG and EA) on the surface [31,32]. The stretching vibration bands of the hydroxyl and amine groups are overlapping and result in a broad region between 3000 cm^−1^ and 3750 cm^−1^.

Zeta potential (ZP) measurements were carried out at ambient pH in distilled water (pH: 6). The surface hydroxyl groups are partially found in a deprotonated form, resulting in negative ZP (−5.6 ± 4.3 mV) (Figure 2B). Next to the -OH groups, -NH_2_ functional groups are found on the nanoparticles, which can become protonated when the pH decreases, leading to the appearance of positive charges (-NH_3_^+^). Due to the positive charges formed because of the protonation of the amine groups, negatively charged algae can form an electrostatic interaction with the magnetic nanoparticles.

**Figure 3:**
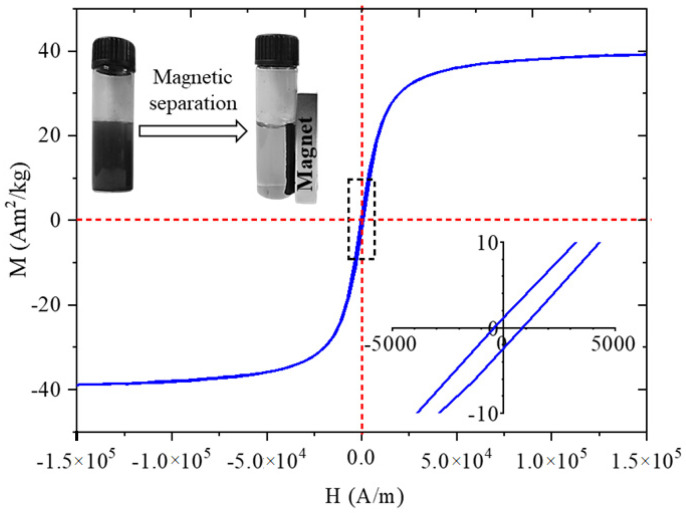
VSM curve and picture of the magnetic separation of the MgFe_2_O_4_-NH_2._ (The inset in the bottom right corner provides an enlarged view of the dashed line section.)

In order to characterize the magnetic behaviour of the amine-functionalized MgFe_2_O_4_ sample, a VSM measurement was conducted at room temperature. The magnetic saturation (Ms) of sample was 39 Am^2^/kg at 150,000 A/m magnetic field (Figure 3). In the case of MgFe_2_O_4_ nanoparticles, similar Ms values (Ms: 12–32 Am^2^/kg) have been reported in other studies [33,34,35]. Our Ms value is higher than the reported Ms value of 27 Am^2^/kg at room temperature for bulk magnesium ferrite [36]. On the magnetization curve, a narrow hysteresis loop with low coercivity (Hc: 955 A/m) and low remanent magnetization (Mr: 1.3 Am^2^/kg) is visible, which are relatively small values that indicate the soft magnetic nature of the particles synthesized at room temperature [37]. Narrow hysteresis loops also indicate that the prepared samples can be easily demagnetized, as clearly shown in the photograph in Figure 3.

### 2.2. Results of the Cobalt Adsorption Tests Using Chlorella vulgaris

After the addition of the cobalt stock solution, the culture Co^2+^ was rapidly removed by the *C. vulgaris* cells. The initial concentration of Co^2+^ in the medium was 63.6 mg/L, which decreased to 2.49 mg/L within 30 min. This represents a removal efficiency of 96.44%. The Co^2+^ concentration in the medium continued to decrease, and after ten hours of cobalt addition, 1.01mg/L Co^2+^ was detected in the cell-free supernatant, giving a removal efficiency of 98.78%.

**Figure 4:**
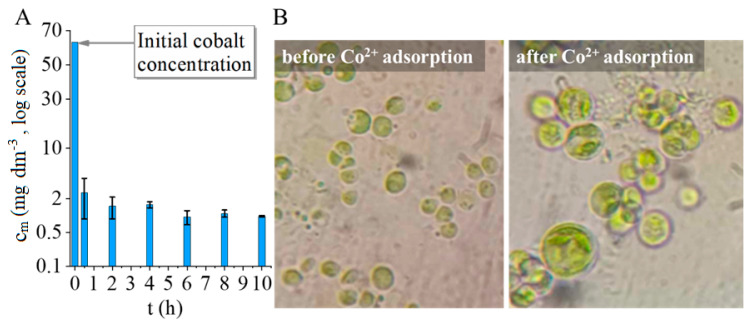
Cobalt adsorption by *Chlorella vulgaris*, cobalt concentration on logarithmic scale vs. contact time (**A**); optical microscopic images from the algae before and after cobalt adsorption (**B**) (1000× magn., oil immersion).

Chlorella cells were observed by light microscopy before and after cobalt treatment, which revealed severe deterioration of cell morphology. Cells became extremely swollen, and a lot of cells showed necrotic morphology with vanished cytoplasm and cell debris (Figure 4).

**Figure 5:**
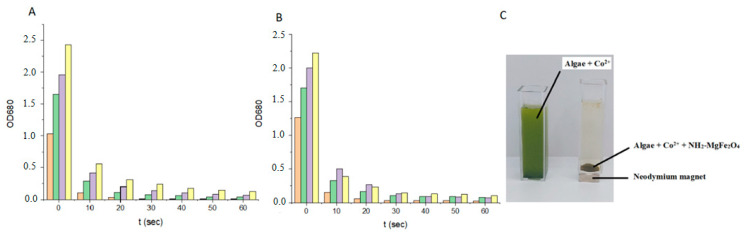
Magnetic separation of normal Chlorella vulgaris (**A**) and cobalt-adsorbed *Chlorella vulgaris*, intensity variation in UV-VIS spectra at different times (**B**), images from algae before and after magnetic separation (**C**).

Under the influence of the magnetic field, the algal biomass bound to the MgFe_2_O_4_ magnetic nanoparticles is completely separated from the nutrient solution within 60 s. There is no significant difference between the separation rate of cobalt-adsorbed algal biomass and normal biomass. For normal algal biomass at a concentration of 3.6 g/L, the optical density at 680 nm decreased from 2.432 to 0.123, representing 94.9% HE%, and for cobalt-adsorbed algal biomass, the optical density decreased from 2.226 to 0.102, representing 95.4% HE%. Magnetic separation of algal biomass of different concentrations is achieved with high efficiency in 60 s, despite the low concentration of magnetizable nanoparticles compared to the concentration of algal suspensions. The optical density at 680 nm of the algal biomass decreases from 2.432 to 0.111 for the highest concentration algae suspension, with 95.4% of the biomass harvested. The optical density of the algal biomass decreases from 1.031 to 0.008 for the lowest concentration algae suspension, with 99.2% HE% (Figure 5). It is expected that the separation efficiency of the higher concentration algal suspension would increase with the concentration of the nanoparticles.

**Figure 6:**
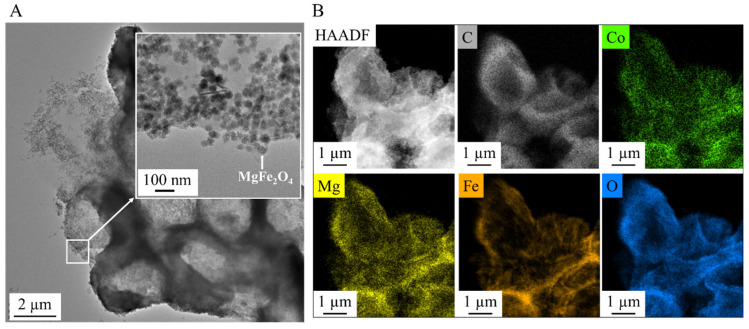
TEM pictures of the algae and the nanoparticles (**A**) and element maps of the algae after cobalt adsorption and magnetic separation (**B**).

We have performed electron microscopy of algae that were magnetically separated from the aqueous medium after cobalt adsorption using magnesium ferrite nanoparticles. The TEM image clearly shows the MgFe_2_O_4_ magnetic nanoparticles evenly distributed on the surface of the algae (Figure 6A). The magnesium ferrite particles with bright and sharp contrast are clearly visible on the high-angle annular dark-field (HAADF) image (Figure 6B). The carbon sign on the elemental maps clearly shows the location of the algae, which have a uniform distribution of adsorbed cobalt ions on their surface. The elemental maps also identify magnesium, iron, and oxygen, which are components of magnesium ferrite. By comparing the positions of magnesium and iron on the elemental maps, it can be observed that magnesium is also enriched where no sign of iron is visible. This can be explained using magnesium sulphate as a nutrient source during algal growth.

## 3. Discussion

This study successfully demonstrates the synthesis and functionalization of amine-modified magnesium ferrite (NH_2_-MgFe_2_O_4_) nanoparticles and their application in the removal of cobalt ions from aqueous solutions using Chlorella vulgaris. The synthesized nanoparticles consisted of aggregated spherical structures composed of primary particles with diameters ranging from 7 to 10 nm. Fourier transform infrared spectroscopy (FTIR) confirmed the presence of -NH_2_ and -OH functional groups, crucial for surface reactivity.

Zeta potential measurements at neutral pH revealed a slightly negative charge (−5.6 ± 4.3 mV), indicating the partial deprotonation of surface hydroxyl groups. However, at lower pH levels, protonation of the -NH_2_ groups leads to positive surface charges, facilitating electrostatic interactions with negatively charged algae. This pH-dependent surface chemistry aligns with previously reported studies that emphasize the critical role of surface functional groups and solution chemistry in nanoparticle–alga interactions [38].

The nanoparticles exhibited strong magnetic properties, with a saturation magnetization (Ms) of 39 Am^2^/kg at 150,000 A/m, confirmed by vibrating sample magnetization (VSM). This property was key to the efficient separation of algae, which bound to the nanoparticles through electrostatic interactions. The algae exhibited high cobalt adsorption efficiency, reducing the Co^2+^ concentration from 63.6 mg/L to 2.49 mg/L within 30 min, achieving a removal efficiency of 96.44%, which further increased to 98.78% after 10 h. These values are significantly higher than many biosorption systems reported in the literature, which typically show lower efficiency or require longer equilibrium times [17]. When exposed to a magnetic field, the algal biomass attached to the MgFe_2_O_4_ magnetic nanoparticles was separated from the nutrient solution with an efficiency of 94.9–99.2% within 60 s. It is important to note that no significant difference was observed in the separation performance of cobalt-adsorbed versus unmodified biomass, suggesting minimal interference of metal binding with magnetic responsiveness. The TEM analysis confirmed the uniform distribution of nanoparticles on the algae surfaces after magnetic separation, thereby supporting the hypothesis that algae can simultaneously adsorb heavy metal ions and nanoparticles.

These findings highlight the synergy between functionalized magnetic nanomaterials and biological systems in developing hybrid technologies for environmental remediation. The use of Chlorella vulgaris, a biocompatible and renewable biosorbent, in combination with reusable magnetic nanoparticles offers a sustainable and scalable approach for heavy metal removal from industrial effluents, particularly in contexts involving battery wastewaters.

While the results are promising, several limitations should be acknowledged. The experiments were conducted under controlled laboratory conditions using synthetic cobalt solutions. Future studies are needed in real wastewater matrices, which typically contain complex mixtures of competing ions and organic contaminants that may influence adsorption and separation efficiency. Additionally, the long-term stability and reusability of the nanoparticle–algae composite system remain to be assessed.

It is also important to consider scalability. Magnetic separators are already employed in a range of industrial applications, including mining, metallurgy, food processing, and wastewater treatment [39]. These systems—such as high-gradient magnetic separators (HGMS) and wet drum separators—commonly operate at magnetic field strengths between 0.1 and 1.5 Tesla, enabling effective removal of magnetic or paramagnetic particles from high-throughput liquid streams [40]. Adapting such technologies to bio-nanocomposite systems will require further engineering development, particularly for large-scale or continuous-flow applications.

In conclusion, this study establishes a novel and efficient biosorption–magnetic separation strategy using NH_2_-MgFe_2_O_4_ nanoparticles and *Chlorella vulgaris*, opening avenues for advanced, eco-friendly wastewater treatment solutions.

## 4. Materials and Methods

### 4.1. Materials

The amine-functionalized magnesium ferrite nanoparticles were synthesized from magnesium nitrate hexahydrate, Mg(NO_3_)_2_·6H_2_O, MW: 290.79 g/mol (ThermoFisher GmbH, D-76870 Kandel, Germany) and iron(III) nitrate nonahydrate, Fe(NO_3_)_3_·9H_2_O, MW: 404.00 g/mol (VWR Int. Ltd., B-3001 Leuven, Belgium). Ethylene glycol, HOCH_2_CH_2_OH, (VWR Int. Ltd., F-94126 Fontenay-sous-Bois, France) was used as dispersion media. For coprecipitation and the functionalization of the ferrites, monoethanolamine, NH_2_CH_2_OH (Merck KGaA, D-64271 Darmstadt, Germany), and sodium acetate, CH_3_COONa (ThermoFisher GmbH, D-76870 Kandel, Germany) were used.

### 4.2. Synthesis of the Amine-Functionalized Magnesium Ferrite Nanoparticles

The synthesis of the magnetic particles was conducted in accordance with the method established by Nonkumwong et al. [41]. Sodium acetate (15 mmol) was dissolved in ethylene glycol (10 mL) and heated to 100 °C and continuously stirred. Magnesium (II)-nitrate (1 mmol) and iron(III)-nitrate (2 mmol) precursors were solved in 50 mL ethylene glycol; after complete dissolution, the two solutions were combined. The mixture was stirred for 30 min, and then ethanolamine (4 mL) was added. The solution was heated to 200 °C and refluxed for 12 h with continuous stirring. The dispersion was cooled down to room temperature, and then separated by magnetic field use of Nd magnet. The solid phase was washed with distilled water several times. Finally, the ferrite was dried at 85 °C overnight.

### 4.3. Cobalt Adsorption Tests

The *Chlorella vulgaris* strain was obtained from the Advanced Materials and Intelligent Technologies Higher Education and Industrial Cooperation Centre at the University of Miskolc.

*C. vulgaris* strain was maintained in modified “endo” medium [42] containing KNO_3_ 3 g/L (Merck KGaA, D-64271 Darmstadt, Germany), KH_2_PO_4_ 1.2 g/L (Merck KGaA, D-64271 Darmstadt, Germany), MgSO_4_·7H_2_O 1.2 g/L (VWR Int. Ltd., B-3001 Leuven, Belgium), citric acid 0.2 g/L (ThermoFisher GmbH, D-76870 Kandel, Germany), FeSO_4_·7H_2_O 0.016 g/L (ThermoFisher GmbH, D-76870 Kandel, Germany), CaCl_2_·2H_2_O 0.105 g/L (VWR Int. Ltd., B-3001 Leuven, Belgium), and trace element stock solution 1 mL/L. For trace elements a stock solution was prepared containing Na_2_EDTA 2.1 g/L (Merck KGaA, D-64271 Darmstadt, Germany), H_3_BO_3_ 2.86 g/L (ThermoFisher GmbH, D-76870 Kandel, Germany), ZnSO_4_·7H_2_O 0.222 g/L (Merck KGaA, D-64271 Darmstadt, Germany), MnCl_2_·4H_2_O 1.81 g/L (Merck KGaA, D-64271 Darmstadt, Germany), Na_2_MoO_4_·2H_2_O 0.021 g/L (VWR Int. Ltd., B-3001 Leuven, Belgium), and CuSO_4_·5H_2_O 0.07g/L (Merck KGaA, D-64271 Darmstadt, Germany). After the solution of all components of the medium, the pH was adjusted to 6.5 ± 0.2 with phosphate buffer and the temperature was controlled at 24 °C.

For growing *C. vulgaris* a glass tubular air lift photo-bioreactor system was constructed, which consisted of Hailea V20 membrane compressor (Guangdong Hailea Group Co., Ltd., Shenzhen, Guangdong, China) with a capacity of 20 L/min air flow with electric power 15W. The reactor system contained 9 pieces of glass vessel with a volume of 500 mL. The air flow rate was 4.5 L/min in each reactor vessel. Reactors were illuminated with. LED lamps with a light intensity of 3000 lumen (ELMARK LYRA15 LED floodlight, 15 W, RGB, IP65, Shenzhen, China). The reactors were inoculated with 4.2 mg/L concentrated *Chlorella v.* culture at a 1% inoculation rate. The initial biomass concentration at inoculation was 0.9 mg/L (=0.5 ± 0.1 OD680) in the reactor vessels. Cell growth was followed by measuring OD680 values by UV-6300PC Doublebeam Spectrophotometer (VWR International, Radnor, PA, USA) of the samples by 24 h.

A cobalt solution (Co(NO_3_)_2_·6H_2_O, Merck KGaA, D-64271 Darmstadt, Germany) with a concentration of 60 mg/L is added to the bioreactors after 24 h of cultivation. Samples of 3 mL were taken from the cultures after 30 min of cobalt addition and after 2, 4, 6, 8, and 10 h. A solution of cobalt at a concentration of 60 mg/L was selected as it represents the toxicity limit for *Chlorella vulgaris* [43].

For Co^2+^ analysis cell-free supernatant was prepared by centrifugation of cultures in Eppendorf tubes at 9000× *g* for 10 min. Supernatants in the amount of 1 mL were transferred to 15 mL Falcon tubes and diluted to 10 mL with ultrapure water.

### 4.4. Magnetic Separation Tests of the Cobalt Adsorbed Algae

In the experiments, the time and efficiency of magnetic separation were compared between cobalt-adsorbed algal biomass and normal algal biomass under different algal concentrations. Concentrated *Chlorella v.* culture containing 4.2 mg/L dry biomass was diluted. Different concentrations of algal suspension were used—3.6 mg/L (=2.0 ± 0.1 OD680), 2.7 mg/L (=1.5 ± 0.1 OD680), 1.8 mg/L (=1.0 ± 0.1 OD680), and 0.9 mg/L (=0.5 ± 0.1 OD680)—while the concentration of NH_2_-Fe_2_O_4_ solution was fixed at 0.511 g/L. An amount of 100 cm^3^ algal suspension of different concentrations was mixed with 0.511 g/L of NH_2_-MgFe_2_O_4_ nanoparticles for 10 min using the air bubbling compressor by Hailea V20 membrane compressor with a capacity of 20 L/min air flow with electric power 15 W. An additional 100 cm^3^ algal suspension of different concentrations a cobalt solution of 60 mg/L was added, and it was mixed with air bubbling for 30 min. Subsequently, the algal biomass was treated with NH_2_-MgFe_2_O_4_ nanoparticles at a concentration of 0.511 g/L for 10 min using the air bubbling compressor. The pH was adjusted to 6.5 with phosphate buffer, and the temperature was 24 °C.

### 4.5. Characterization Techniques

The morphological characterization and size analysis of the magnetic nanoparticles was conducted by utilizing high-resolution transmission electron microscopy (HRTEM, Talos F200X G2 electron microscope with field emission electron gun, X-FEG, accelerating voltage: 20–200 kV, ThermoScientific, Waltham, MA, USA). For the imaging and selected area electron diffraction (SAED), SmartCam digital search camera (Ceta 16M CMOS camera, ThermoFisher Scientific, Waltham, MA, USA) was used with a high-angle annular dark-field (HAADF) detector. During sample preparation, the aqueous dispersion of the nanoparticles was dropped onto copper grids (Ted Pella Inc., 4595 Redding, CA 96003, USA). For phase analysis of the magnetic nanoparticles, X-ray diffraction (XRD) method and Rietveld refinement were applied. For X-ray diffraction measurements Bruker D8 diffractometer (Cu-Kα source) (Bruker AXS SE, 76187 Karlsruhe, Germany) in parallel beam geometry (Göbel mirror) with Vantec detector was used. The functional groups on the surface of the amine-functionalized magnesium ferrite were examined with Fourier transform infrared spectroscopy (FTIR), using a Bruker Vertex 70 spectroscope (Bruker Optics GmbH & Co. KG, 76275 Ettlingen, Germany). The measurements were conducted in transmission mode, while the sample preparation involved the use of palletization in spectroscopic potassium bromide. (10 mg ferrite sample was measured to 200 mg KBr). The zeta potential of the nanoparticles was measured based on electrophoretic mobility by applying laser Doppler electrophoresis with a Malvern Zetasizer Nano ZS equipment (Malvern Panalytical, Malvern, UK). The specific surface area (SSA) of the samples was measured by carbon dioxide adsorption–desorption experiments at 273 K based on Dubinin-Astakhov (DA) method (with Micromeritics ASAP 2020 equipment, Micromeritics Instrument Corporation, Norcross, GA, USA). To measure the saturation magnetization (Ms), remained magnetization (Mr), and the coercivity (Hc) of the samples was carried out with vibrating-sample magnetometer (VSM) system based on a water-cooled Weiss-type electromagnet (self-developed magnetometer by University of Debrecen). For the VSM test, the sample was pelletized with mass of 20 mg. The magnetization (M) was measured as a function of magnetic field (H) up to 150,000 A/m field strength at room temperature.

The concentration of cobalt ions in the supernatant was determined by inductively coupled plasma atomic emission spectrometry (ICP-AES). The ICP instrument was a Varian-make 720 ES type simultaneous, multielement spectrometer with axial plasma view (Agilent Technologies, Mulgrave, Victoria, Australia). The parameters of the analyses were the following: running frequency of the RF generator: 40[MHz]; sample introduction device: V-groove nebulizer with Sturman–Masters spray chamber; sample uptake: 2.1 cm^3^/min; length of signal integration: 8 s; number of reading: 3/sample. The calibration of the instrument was performed using a solution series prepared from a 1000 mg/dm^3^ monoelement stock cobalt solution (Merck KGaA, D-64271 Darmstadt, Germany) 

Co^2+^ removal efficiency was calculated by the following equation:

$$\mathrm{Removal}\;\mathrm{efficiency}\;\%=\;\frac{{{[Co}^{2+}]}_{t0}-{\left[{Co}^{2+}\right]}_{t1}}{{{[Co}^{2+}]}_{t0}}\times 100$$

[Co^2+^]_t0_ = initial concentration of Co^2+^ given in mg/L,

[Co^2+^ ]_t1_ = Co^2+^ concentration of the given time point after cobalt addition of the cell free supernatant.

To determine the efficiency of nanoparticles to bind to the surface of cobalt-adsorbed algal cells and normal algal cells, we measured their sedimentation rate in a magnetic field. An amount of 4 mL of the algal suspension was measured in a special cuvette with a 10 mm × 10 mm × 3 mm N45 neodymium magnet glued to the bottom. The cuvette with the magnet was placed in the spectrophotometer cuvette holder and the sedimentation rate was measured by the change in optical density over time. The change in optical density was followed by measuring at 680 nm by UV1100/UV-1200 Doublebeam spectrophotometer (Frederiksen Scientific A/S, DK-6870, Ølgod, Dania).

The harvesting efficiency (HE%) of the process was calculated from equation:

$$\mathrm{HE}\;\%=\frac{{\mathrm{OD}}_{0}-{\mathrm{OD}}_{1}}{{\mathrm{OD}}_{0}}\times 100$$

OD_0_ = initial absorbance of the microalgae cultivation at a wavelength of 680 nm,

OD_1_ = absorbance at the same wavelength of the supernatant liquid that separates from the microalgae-particles flocs after the application of the magnetic field.

## Data Availability

The original contributions presented in this study are included in the article. Further inquiries can be directed to the corresponding author(s).

## Abbreviations

HRTEM	High-resolution transmission electron microscopy
FTIR	Fourier transform infrared spectroscopy
SEM	Scanning electron microscopy
VSM	Vibrating sample magnetometry
NMC	Nickel manganese cobalt oxide
NCA	Nickel cobalt aluminum oxide
PDDA	Poly diallyldimethylammonium chloride
PEI	Polyethylenimine
APTES	3-aminopropyl triethoxysilane
SAED	Selected area electron diffraction
HAADF	High-angle annular dark-field
ICP-AES	Inductively coupled plasma atomic emission spectrometry
